# Age Disparities in Prevalence of Anxiety and Depression Among US Adults During the COVID-19 Pandemic

**DOI:** 10.1001/jamanetworkopen.2023.45073

**Published:** 2023-11-30

**Authors:** Sarah Collier Villaume, Shanting Chen, Emma K. Adam

**Affiliations:** 1 School of Education and Social Policy, Northwestern University, Evanston, Illinois; 2 Institute for Policy Research, Northwestern University, Evanston, Illinois; 3 Department of Psychology, University of Florida, Gainesville

## Abstract

**Importance:**

High levels of anxiety and depression were documented shortly after the arrival of the COVID-19 pandemic and were more prevalent in younger adults than in older adults. Knowing whether these age disparities persisted throughout multiple years of the COVID-19 pandemic and identifying associated factors will help guide health policy.

**Objective:**

To investigate age disparities in anxiety and depression during the COVID-19 pandemic.

**Design, Setting, and Participants:**

This cross-sectional study consisted of a nationally representative online survey administered between April 2020 and August 2022 and included US adults who were not incarcerated. Data were analyzed between March and September 2022.

**Exposures:**

The first 27 months of the COVID-19 pandemic included wide variation in infection rates, turbulence in US political and social life, and geopolitical instability. Primary exposures include individuals’ age and economic precarity and pandemic-related events (eg, weekly state-level case counts and individual vaccination status).

**Main outcomes and measures:**

Symptoms of anxiety and depression were assessed via responses to 2-item screeners (Generalized Anxiety Disorder 2-item for anxiety and Patient Health Questionnaire-2 for depression). An individual’s symptoms were identified as clinically elevated if scores exceeded validated thresholds.

**Results:**

This study included 3 028 923 respondents (mean [SD] age, 48.9 [17.0] years; 1 567 603 [51.8%] female). In multiple regression analyses that include state fixed effects and survey-week fixed effects, likely anxiety and depressive disorders among 291 382 (40%) and 238 505 (33%) of adults aged 18 to 39 years, respectively, compared with 357 820 (31%) and 274 534 (24%) of adults aged 40 to 59 years and 225 295 (20%) and 183 695 (16%) adults aged 60 years and older. Levels declined throughout the pandemic period for those aged 40 years and older but remained elevated for younger adults. Analyses identified several associated factors of these age disparities. Younger adults’ anxiety and depression increased more than older adults’ after surges in COVID-19 case counts but decreased less following vaccination against the virus. Additionally, approximately one third of the age gap among individuals with depression and anxiety was attributed to economic precarity, to which younger adults are disproportionately exposed.

**Conclusions and relevance:**

In this cross-sectional study of anxiety and depression during the COVID-19 pandemic, economic precarity was associated with high anxiety and depression among younger adults in the US compared with older adults in the US. These findings suggest a need for greater mental health care and economic policies targeted toward younger adults.

## Key Points

**Question:**

Were there age disparities in anxiety and depression during the COVID-19 pandemic?

**Findings:**

In this cross-sectional study of 3 028 923 US adults, anxiety and depression were significantly higher among adults aged 18 to 39 years (40% and 33%, respectively) compared with adults aged 40 years and older (31% and 24%, respectively). Greater economic precarity and greater reactivity to changing case counts among younger adults were associated with this age disparity.

**Meaning:**

These findings suggest that more than one-third of young adults had anxiety or depression during the COVID-19 pandemic; less favorable economic conditions and responses to social upheaval may have contributed to young adults’ worse mental well-being.

## Teaser

This cross-sectional study investigates age disparities in anxiety and depression throughout the COVID-19 pandemic.

## Introduction

The years from 2020 through 2022 were difficult, due to the combination of the COVID-19 pandemic and a series of national and global events that impacted people in the US and around the world. From waves of COVID-19 morbidity and mortality to protests against police violence, climate catastrophes, and mass shooting events, the COVID-19 pandemic era can be thought of as a period of chronic stress, punctuated with acute stressors. Both chronic and recent stress exposure are known factors of anxiety and depression symptoms and disorders.^1,2,3^ Indeed, high levels of anxiety and depression were identified early in the COVID-19 pandemic, with reports of up to 6-fold^4,5,6,7,8^ increase from prior-year levels. Most published studies focused on the initial months after the pandemic’s onset; more recent data allows us to examine whether high levels of depression and anxiety persisted across multiple years of the COVID-19 pandemic.

A concerning age disparity in symptoms and disorders of depression and anxiety emerged in the decade prior to the COVID-19 pandemic, with adolescents and young adults showing higher levels of depression and anxiety than older adults.^9,10,11^ However, it is not clear whether mental health worsened early in the pandemic primarily among younger adults or throughout the adult age distribution^12^ (eFigure 1 in Supplement 1). It is also unclear whether the age disparity widened or narrowed throughout the pandemic period and what factors might account for the age disparity or time trends.

The present study examines age disparities in symptoms of anxiety and depression across the first 2 years of the COVID-19 pandemic. To our knowledge, it is the first to examine symptoms of anxiety and depression in nationally representative data collected more recently than June 2021. We analyzed more than 3 million responses to the Household Pulse Survey (HPS) from April 2020 through August 2022, alongside weekly state-level data on COVID-19 cases and deaths.^13^

Our objective was to examine whether levels of depression and anxiety in younger adults are higher than for those in middle and older adulthood and to determine whether this disparity spanned the first 2 years of the pandemic period. We investigated whether pandemic- and nonpandemic-related stress exposures measured in the HPS differed by age group and were associated with the age disparities in mental health observed. Exposures included younger adults’ economic circumstances and responses to pandemic case counts and vaccine availability. Finally, we used a decomposition analysis to quantify the proportion of the age disparity that is explained, statistically, by differences in stress exposure between age groups, vs differences in their responses (vulnerability) to these stressors. We hypothesized that anxiety and depression prevalence would be higher for younger adults than their older counterparts at the beginning of the pandemic and would fall for all age groups as vaccines became available.

## Method

This cross-sectional study followed the Strengthening the Reporting of Observational Studies in Epidemiology (STROBE) reporting guideline. The institutional review board at Northwestern University confirmed this study is not human participant research and waived the need for informed consent and review.

### Data Source

The US Census Bureau developed the HPS early in the COVID-19 pandemic. Forty-eight surveys were administered online to a mean (SD) of approximately 63 000 (13 551) respondents per survey between April 2020 and August 2022 (mean [SD], 18 [11] days) (eTable 1 in Supplement 1). The median (IQR) response rate was 6.4% (4.3%-7.2%) and increased slightly in later surveys. We excluded any response missing both anxiety and depression data (474 826 [13%]). While the HPS surveyed a small proportion of respondents (158 644 [5%]) multiple times, our analytic sample included only 1 response per person, retaining the first response with complete anxiety or depression data.

### Measures

#### Outcome Variables and Covariates

Respondents completed 2-item screeners, the Generalized Anxiety Disorder Screener (GAD-2)^14^ for anxiety and the Patient Health Questionnaire (PHQ-2)^15^ for depression. Items index how often symptoms were experienced in the past 2 weeks (0, not at all; 3, nearly every day). Responses were summed to create an anxiety score and a depression score, with totals of 3 or greater interpreted as clinically significant.^14,15,16^ Analyses adjusted for respondent sex, age, race or ethnicity, education level, and income (eTable 2 in Supplement 1). Survey respondents were asked to self-identify their ethnicity and race, and responses were then aggregated and recoded into a combined ethnicity and race variable (White or racial and ethnic minority). This study investigated factors of inequality in Americans’ experiences of the COVID-19 pandemic. Given the US history of chattel slavery and the ongoing harms perpetuated against members of ethnically and racially minoritized groups—and their implications for health and well-being—we had reason to hypothesize that group membership might be associated with other factors examined in this study.

#### Exposures

COVID-19 cases and deaths at the state level were obtained from the US Department of Disease Control and Prevention Case Surveillance data set.^13^ Prior-week counts were computed by subtracting each state’s cumulative cases (deaths) from its prior-week figure. Values are expressed in terms of cases per 100 and deaths per 1000 residents, with state population obtained from the 2020 census. All subsequent exposures were measured at the individual level.

COVID-19–related HPS survey measures included 2 questions asking if they had ever been diagnosed with COVID-19 (1, yes; 0, no) and if they had received at least 1 dose of a vaccination against COVID-19 (1, yes; 0, no). Both questions were added to the HPS in January of 2021 (eTable 1 in Supplement 1).

### HPS Measures of Economic Precarity

Several variables were used to assess the extent to which a respondent is economically precarious. We use this term to refer to the uncertainty of a person’s position, which may predispose them to greater risk exposure during turbulent times. Among potential sources of precarity assessed in the HPS, an indicator variable was created to capture whether a respondent resides in a home that is owned rather than rented. An item that asked whether respondents worked for pay in the past 7 days was used as a measure of recent employment. A question about income loss initially referred to income loss experienced since the start of the COVID-19 pandemic and was updated in April 2021 to refer to the past 4 weeks. Models included a separate indicator variable for each version of this question. An alternative approach to measuring economic precarity used a composite measure (a risk score) that assigned a score of 1 for each source of precarity a respondent reported: educational attainment below a bachelor’s degree, annual household income below $35 000, living in a home that is not owned, or any income loss.

### Statistical Analysis

This data set was analyzed as panel data with state fixed effects and survey-week fixed effects.^17^ Regression models first examined demographic differences in the prevalence of anxiety and depression. Our second set of analyses asked whether younger adults experienced more stressful conditions than those in middle or older adulthood. Here, we estimated each stressor of interest from age category in a separate regression model. The stressors examined were directly associated with the pandemic as well as economic precarity or household composition.

Next, we considered whether such stressors might be more strongly associated with anxiety or depression for young adults vs middle adults. Regression models estimated anxiety or depression from each stress exposure of interest (eg, home ownership) and the interaction between that stressor and a variable for young adulthood (home ownership under age 40 years).

Finally, we used the Blinder-Oaxaca decomposition to quantify how explanatory variables account for anxiety and depression differently between adults aged 18 to 39 years and aged 40 to 59 years. This method fits a regression model separately for each group and then separates the difference in average levels of the outcome into 2 components. The first, the endowment effect, reflects the change in the outcome that would, statistically, be expected if the 2 groups were exposed to the same conditions (ie, if 1 group took on the covariate distribution of the other).^18^ The second, the coefficient effect, captures differences in the association of explanatory variables. This portion is attributed to age group differences in vulnerability to stressful conditions (and unmeasured variance, including error).^19^

Analyses were conducted using Stata/MP version 17.0 (StataCorp), with sampling weights that adjust for nonresponse and yield nationally representative estimates.^20^ An a priori threshold of *P* <.05 was set for statistical significance. All tests were 2-sided.

## Results

We analyzed data from 3 028 923 respondents (mean [SD] age, 48.9 [17.0] years; 1 567 603 [51.8%] female; 1 926 300[63.6%] non-Hispanic White; 498 145 [16.4%] Hispanic or Latino; and 333 805 [11.0%] non-Hispanic Black). Descriptive statistics appear in eTables 2 to 3 in Supplement 1.

### Disparities in Anxiety and Depression During the COVID-19 Pandemic 

#### Age Disparities in Prevalence

**Figure 1.**
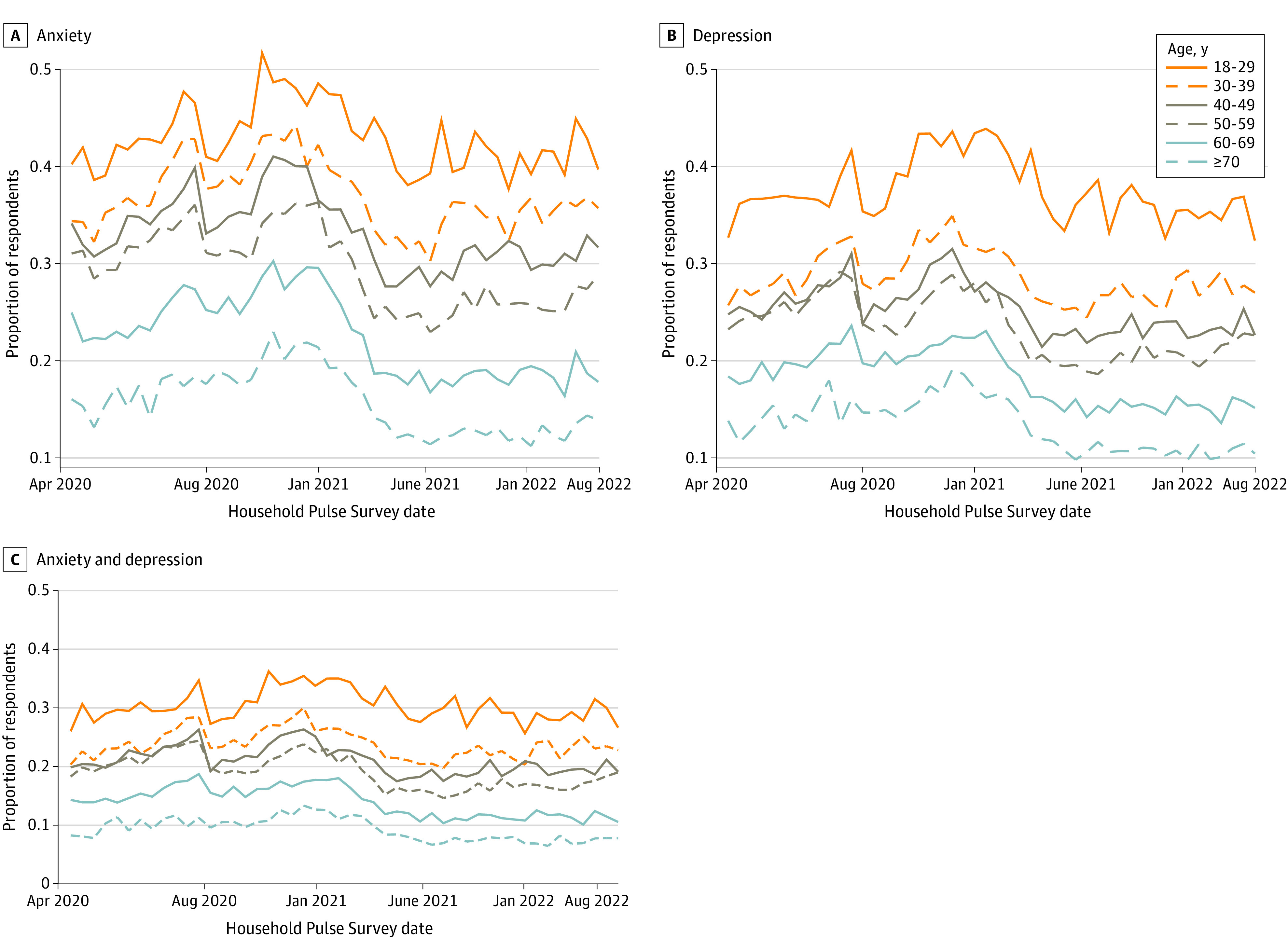
Anxiety and Depression by 10-Year Age Category This figure shows the proportion of respondents in each 10-year age group whose scores exceed thresholds for clinically significant (A) anxiety, (B) depression, or (C) both anxiety and depression. Plots reflect population-weighted descriptive statistics (not regression-adjusted values). Calculations were conducted using data from Household Pulse Surveys April 2020 to August 2022.^21^

Anxiety and depression were high throughout the study period, especially for young adults (Figure 1). On average throughout the pandemic, clinically significant anxiety was exhibited by 40.4% (95% CI, 39.5%-41.3%) (eTable 4 in Supplement 1) and clinically significant depression by 36.3% (95% CI, 35.4% to 37.1%) of respondents aged 18 to 29 years. The prevalence of anxiety was approximately 4 percentage points lower for each 10-year age group (*P* <.001); depression prevalence was also lower for older age groups, with smaller gaps observed between some age categories (Figure 1).

The remainder of this article presents results by 20-year age category, comparing young adults (aged 18 to 39 years) to those in middle adulthood (aged 40 to 59 years). Respondents aged 60 years and older were excluded because many are not among the working age population and likely experienced pandemic-related income disruptions differently than younger US adults.

Anxiety and depression scores and prevalence appear by 20-year age category in eTable 3 in Supplement 1. Scores were higher for anxiety and depression among those in young adulthood compared with middle adulthood (mean [SD] anxiety score: 2.44 [2.11] vs 2.00 [2.06] and mean [SD] depression score, 2.05 [2.00] vs 1.62 [1.87], respectively; *P* <.001) (eTable 3 in Supplement 1).

#### Other Demographic Differences in Prevalence

**Figure 2.**
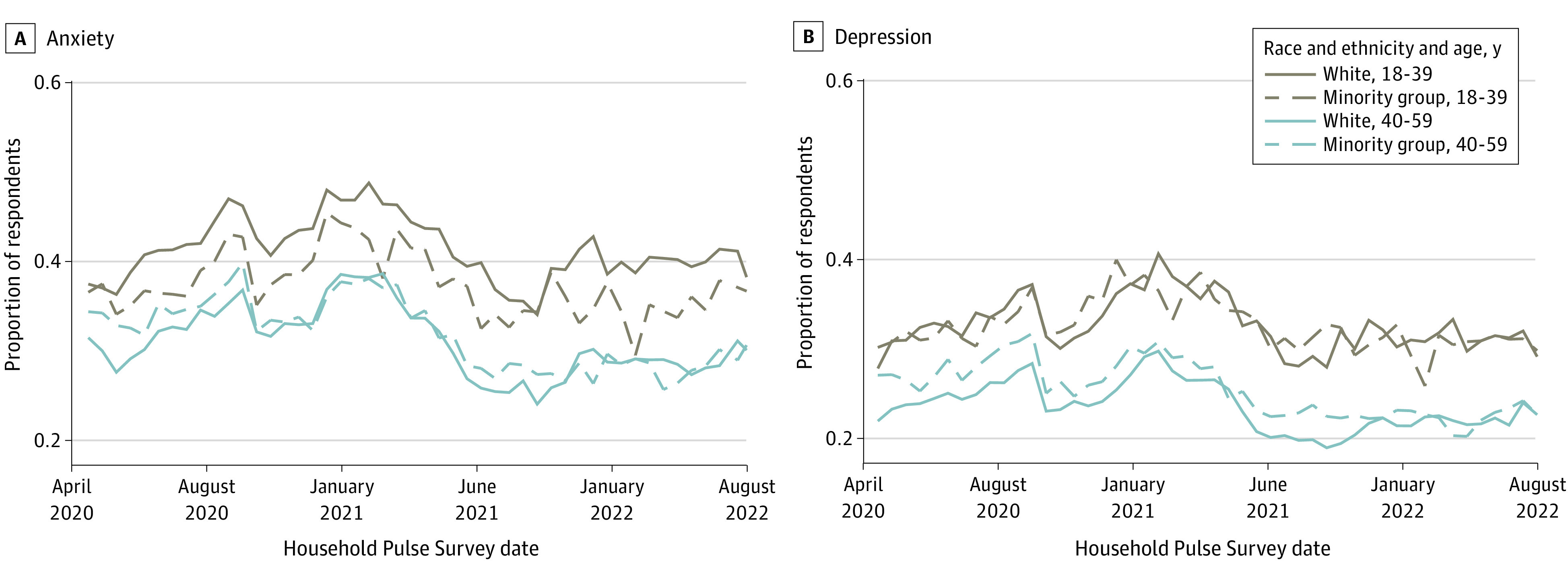
Anxiety and Depression for White Respondents and Individuals From Racially and Ethnically Minoritized Groups, by Age Group Calculations were conducted using data from Household Pulse Surveys April 2020 to August 2022.^21^ This figure shows that White respondents exhibit a larger spread in anxiety and depression than individuals from racially and ethnically minoritized groups, with the highest levels of anxiety and depression among White respondents aged 18 to 39 years and the lowest levels among White respondents aged 40 to 59 years (whereas prevalence for individuals from racially and ethnically minoritized groups tends to fall between these extremes for both age groups). Plots reflect population-weighted descriptive statistics (not regression-adjusted values). Minority group indicates individuals from racially and ethnically minoritzed groups.

**Figure 3.**
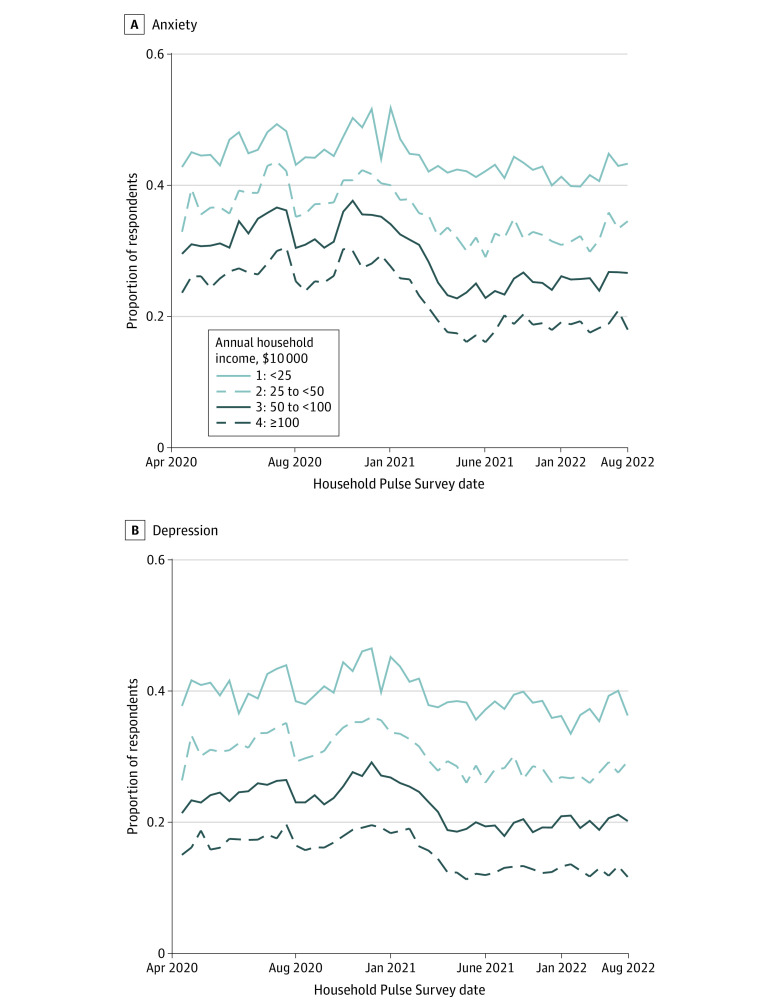
Anxiety and Depression by Household Income This figure shows the proportion of respondents with clinically elevated (A) anxiety and (B) depression over the pandemic period, grouped by annual household income level. Plots reflect population-weighted descriptive statistics (not regression-adjusted values). Calculations were conducted using data from Household Pulse Surveys April 2020 to August 2022.^21^

Anxiety and depression were significantly more prevalent among females than males (eTable 4 in Supplement 1). Figure 2 shows that the age disparity in anxiety and depression was present for both White individuals and individuals from racially and ethnically minoritized groups; it was significant for both groups but larger for White individuals (eTable 4 in Supplement 1). Individuals with below a bachelor’s degree (BA) had significantly higher levels of anxiety and depression than those with a BA or above (eTable 4 in Supplement 1). Anxiety and depression prevalence also followed an income gradient. As Figure 3 shows, lower household incomes were associated with significantly greater prevalence of anxiety and depression (eTable 4 in Supplement 1).

#### Overlap Between Anxiety and Depression

Anxiety and depression are often comorbid conditions.^22^ In this sample, more than half of respondents with high anxiety scores also had high depression scores (380 820 of 634 204 [60.1%] of those with any anxiety have both anxiety and depression) and more than 80% of those with high depression scores also had high anxiety scores (380 820 of 457 836 [83.2%] of those with any depression had both anxiety and depression). Figure 1 and eFigure 2 in Supplement 1 depict patterns of cooccurrence by 10-year and 20-year age groups, respectively.

#### Changes in Age Disparities Over Time

The highest levels of anxiety and depression were observed early in the pandemic, with a peak in late 2020 and a pronounced decline in anxiety (and, to a lesser degree, depression) beginning in early 2021. The peak roughly coincided with the fall 2020 surge in COVID-19 cases—and the decline followed the availability COVID-19 vaccination (Figure 2 and eFigure 3 in Supplement 1). This early 2021 decline was more pronounced for respondents in middle adulthood than for young adults, who exhibited persistently high levels of anxiety and depression. As a result, the age gap widened. For anxiety, it increased approximately 33% from the April 2020 to January 2021 period to the January 2021 to August 2022 period from 7.0 (95% CI, 6.51-7.46) percentage points to 9.3 (95% CI, 8.91-9.72) percentage points. For depression, it increased more than 20%, from 7.5 (95% CI, 7.08-8.00) percentage points to 9.1 (95% CI, 8.74-9.53) percentage points, throughout the same period.

### Exposure to More Stressful Conditions by Age Group

Adults aged 18 to 39 years reported lower household incomes (60% the odds of earning $100 000 or more) (eTable 2 in Supplement 1) and lower rates of living alone than those in middle adulthood. They had approximately half the odds of residing in an owned home compared with those aged 40 to 59 years. Younger adults also had higher scores on the economic risk composite. However, the 2 groups reported similar rates of pandemic-related income loss and recent employment. Regarding COVID-19 exposure, adults aged 18 to 39 years were more likely than those aged 40 to 59 years to report that they have been diagnosed with COVID-19 (OR, 1.04; 95% CI, 1.02-1.06) and less likely to report vaccination against it (OR, 0.76; 95% CI, 0.74-0.77).

### Association of Exposure to Stress With Anxiety and Depression

#### Pandemic Severity

**Figure 4.**
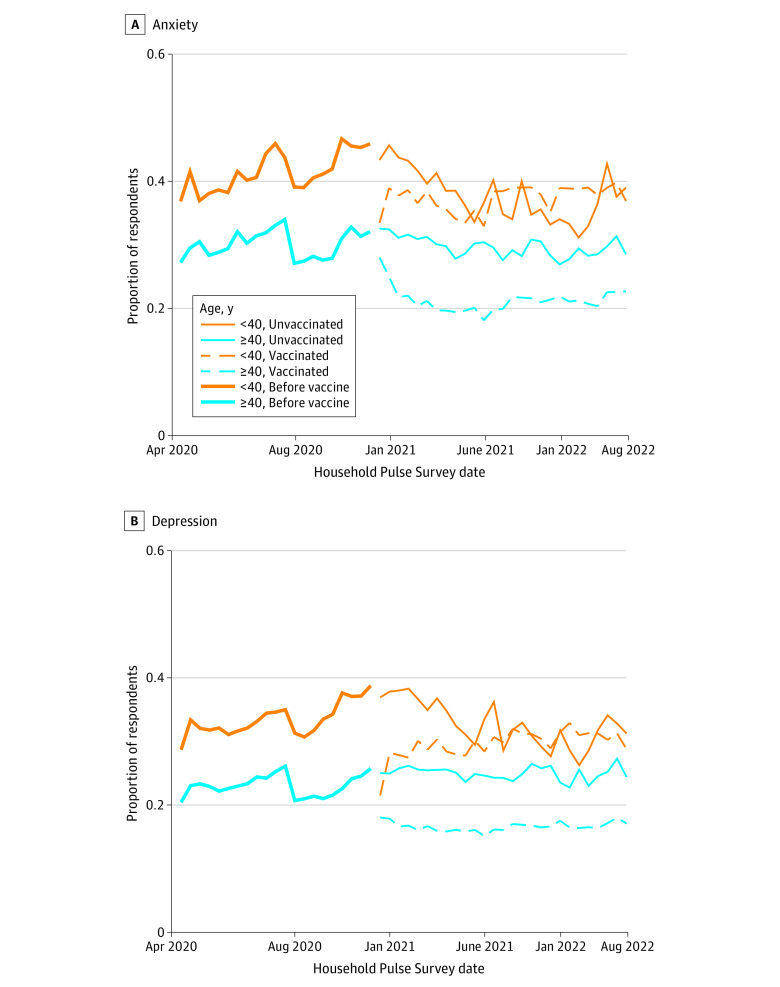
Anxiety and Depression by Vaccination Status and Age Group This figure depicts the proportion of respondents with clinically elevated (A) anxiety and (B) depression over the pandemic period. The thicker lines correspond to the time period before vaccination was available in the US. The finer lines distinguish between survey respondents who indicated that they have received at least 1 dose of a vaccine (dashed line) or have not yet been vaccinated (solid line). Plots reflect population-weighted descriptive statistics (not regression-adjusted values). Calculations were conducted using data from Household Pulse Surveys April 2020 to August 2022.^21^

COVID-19 case counts were more strongly associated with anxiety and depression for younger adults than for older adults (eTable 5 in Supplement 1). However, vaccination against the virus was associated with greater improvements in mental well-being of adults aged 40 years or older compared with those aged 18 to 39 years (Figure 4 and eTable 6 in Supplement 1).

#### Economic Precarity

Individuals who were high on the economic risk score in both age groups exhibited high levels of anxiety and depression (eFigure 4 in Supplement 1). There was little age-related difference in mental health among adults experiencing high levels of economic precarity (eTable 7 in Supplement 1).

Several additional specifications were tested to ensure our measurement of economic precarity were not associated with instability with household composition. Results confirmed that mental health was not worse among respondents who live alone or who report residing with children.

### Decomposition Analysis

Our final analysis separates the age disparity in anxiety and depression into 2 components: the portion that can be attributed to differences that would, statistically, be estimated to disappear if the younger group had the same observable characteristics as their older counterparts, and the portion that would not.^19^ Results indicate that differences in demographic characteristics, including income, accounted for approximately 20% of the age disparity in anxiety and depression (estimated separately) (eTable 8 in Supplement 1). This estimate would translate to an estimated 1.8 million fewer young adults in the US exhibiting clinically meaningful symptoms of anxiety or depression. In subsequent models, accounting for home ownership or the economic risk score each increased the proportion explained to more than 30%. In contrast, no reduction in anxiety or depression was estimated if younger adults took on the response of their older counterparts to infection with or vaccination against COVID-19 (eTable 8 in Supplement 1).

## Discussion

Survey responses from more than 3 million US adults suggest that high levels of anxiety and depression among young adults, which were reported in the pandemic’s first year,^4,5,6,8,23^ persisted throughout the years since the onset of the COVID-19 pandemic. We observed the highest levels of anxiety and depression among adults under the age of 40 years. Our analyses offer key insights into factors of this disparity.

Additionally, we found that young adults experience more economic precarity than older adults, including lower household income and lower odds of homeownership.^24^ These types of precarity are not specifically related to the COVID-19 pandemic and, for many young adults, likely predated the pandemic period.^10^

Younger adults showed greater sensitivity to fluctuations in COVID-19 case counts than those aged 40 years and older. This vulnerability may be consistent with a heightened responsivity to stressors that is characteristic of adolescents and young adults.^25^ This predisposition may be accentuated by the chronic and repeated episodic stressors of the pandemic. In contrast, middle adulthood was associated with vulnerability to several other exposures we examined. For instance, greater responsiveness to vaccination against the COVID-19 may help to explain the reduction in anxiety and depression that adults aged 40 years and older experienced beginning in early 2021,^6^ a shift that began around when vaccines became available and persisted through 2022 but was not shared by adults aged 18 to 39 years.

Exposure to economic precarity also accounted for a substantial portion of the age disparity in anxiety and depression. In fact, adults aged 40 years and older who are economically precarious look quite similar to adults aged 18 to 39 years. This tells us that lower levels of anxiety and depression among adults aged 40 years or older may partially reflect the higher levels of economic privilege they have, on average, compared with their younger counterparts.

Decomposition analysis estimated that differences in the demographic and economic conditions to which young adults vs middle adults are exposed account for approximately one third of the age disparity in anxiety and depression. This is the portion of the age disparity that could, in theory, be reduced if younger adults instead experienced the material conditions of their older counterparts. These analyses indicate that approximately one third of the age disparity can be attributed to younger adults’ greater exposure to stressors that undermine their stability.

Across these research questions, we examined a range of potential factors that may be associated with anxiety and depression symptoms observed. We regarded certain stress exposures, like COVID-19 case counts and vaccination, as explicitly pandemic-related and others as more related to economic precarity. This categorization may imply a separation between 2 types of stress that, in reality, would be expected to influence one another. Social and economic privilege would be expected to affect a person’s odds of contracting the virus as well as their access to paid sick days or the ability to work remotely. Accordingly, our interpretation of a result like younger adults’ greater responsiveness to COVID-19 case counts (vs those aged 40 to 59 years) should reflect a possible association with concerns about the virus and concerns about economic stability (for instance, worrying that a virus surge might lead to loss of income). Moreover, even if their own risk of infection was not the primary driver of young adults’ concerns, their symptoms of anxiety and depression could stem from risks the virus poses to other loved ones. Alternatively, their symptoms may follow from other ways that pandemic surges disrupt economic activity or our social fabric (eg, increases in social isolation and the disruption of holiday gatherings or other celebrations). Finally, we cannot rule out the possibility that current events or social stressors contributed to the persistently elevated anxiety and depression observed among young adults. Data were collected across a period that included racial justice protests, mass shootings, the Russian invasion of Ukraine, decades-high inflation, and recurrent climate catastrophes, to name just a few of the period’s destabilizing events. In fact, according to the 2022 Stress in America Survey, many US adults describe a profound sense of loss and grief regarding the COVID-19 pandemic,^26^ alongside worries about household finances and the economy and feelings of unease about the geopolitical events in Ukraine.^26^ These sentiments suggest this period is marked by a sense of upheaval that has left a lasting impact on young adult well-being.

### Limitations

This study has limitations. While repeated cross-sectional surveys can provide a view on population trends throughout the pandemic, they do not follow the same set of individuals over time. This prohibits drawing conclusions about the causal impact of a change in stress exposure over time on an individual’s mental well-being. It is also worth noting that the 2 age groups we compare were surveyed simultaneously; we do not draw conclusions about how the mental health of today’s young adults will change as they get older or even as the COVID-19 pandemic period transitions into a new normal with fewer public health guidelines in place. Our comparisons concern only group differences observed during the COVID-19 pandemic period.

Online surveys like the HPS tend to have lower response rates than telephone surveys, which raises concerns that the people choosing to respond to survey invitations differ meaningfully from those who are recruited but do not respond. This type of measurement error cannot be eliminated with sampling weights.^20^ We recommend relying on the HPS for the types of insights to which it is well-suited; for instance, it offers a large sample size and good temporal resolution across the pandemic. We echo the US Census Bureau’s caution that, as an experimental data product, the HPS should not be compared directly with nationwide prevalence estimates that are derived from other well-established surveys.^20^ Finally, our outcome variables come from screening questions that assess symptoms of anxiety and depression for potential clinical significance; they should not be conflated with the clinical diagnosis of an anxiety or depressive disorder.

## Conclusions

In this cross-sectional study, we document concerning levels of anxiety and depression among young adults that persisted throughout the first 2 years of the COVID-19 pandemic. More than one third of the age-related gap in anxiety and depression was attributed to differences in demographic and economic conditions between young and middle adults in the US. We find that younger adults are sensitive to fluctuations in COVID-19 case levels and speculate that they may show heightened responsiveness to other societal events that have occurred during the pandemic period. While there is more to learn about the factors that contribute to the experience younger US adult have with anxiety and depression in the current context, our findings point to a need for mental health care and economic policies that target the needs of young adults.

## Notes

Supplement 1. **eFigure 1.** Prepandemic Trends in Depressive Symptoms **eTable 1. **Household Pulse Survey Administration Dates **eTable 2. **Descriptive Statistics—Exposures and Covariates **eTable 3. **Descriptive Statistics—Outcome Variables **eTable 4.** Detailed Regression Results **eFigure 2.** Co-occurrence of Anxiety and Depression **eFigure 3.** Anxiety and Depression for White Respondents and Individuals From Racially and Ethnically Minoritzed Groups, by Age Group, Annotated With Current Events **eTable 5.** Impacts of Pandemic Burden on Anxiety and Depression, Overall and for Ages 18 to 39 Years vs Ages 40 Years and Older **eTable 6.** Impacts of Prior COVID-19 Diagnosis and Vaccine Receipt **eFigure 4.** Anxiety and Depression by Economic Precarity Score and Age Group **eTable 7.** Prevalence of Anxiety and Depression by Economic Precarity **eTable 8.** Proportion of Age Disparity Accounted for by Exposure Effect

Supplement 2.
Data Sharing Statement
