# Supplementary material for: Age Disparities in Prevalence of Anxiety and Depression Among US Adults During the COVID-19 Pandemic
Source: JAMA Netw Open. 2023 Nov 30;6(11):e2345073. doi: 10.1001/jamanetworkopen.2023.45073 (PMC10690464; doi:10.1001/jamanetworkopen.2023.45073)
Supplement: Supplement 1. — eFigure 1. Prepandemic Trends in Depressive Symptoms eTable 1. Household Pulse Survey Administration Dates eTable 2. Descriptive Statistics—Exposures and Covariates eTable 3. Descriptive Statistics—Outcome Variables eTable 4. Detailed Regression Results eFigure 2. Co-occurrence of Anxiety and Depression eFigure 3. Anxiety and Depression for White Respondents and Individuals From Racially and Ethnically Minoritzed Groups, by Age Group, Annotated With Current Events eTable 5. Impacts of Pandemic Burden on Anxiety and Depression, Overall and for Ages 18 to 39 Years vs Ages 40 Years and Older eTable 6. Impacts of Prior COVID-19 Diagnosis and Vaccine Receipt eFigure 4. Anxiety and Depression by Economic Precarity Score and Age Group eTable 7. Prevalence of Anxiety and Depression by Economic Precarity eTable 8. Proportion of Age Disparity Accounted for by Exposure Effect [file jamanetwopen-e2345073-s001.pdf]

Supplemental Online Content

Collier Villaume S, Chen S, Adam EK. Age disparities in prevalence of anxiety and depression among US adults during the COVID-19 pandemic. *JAMA Netw Open*. 2023;6(11):e2345073. doi:10.1001/jamanetworkopen.2023.45073

eFigure 1. Prepandemic Trends in Depressive Symptoms
eTable 1. Household Pulse Survey Administration Dates
eTable 2. Descriptive Statistics—Exposures And Covariates
eTable 3. Descriptive Statistics—Outcome Variables
eTable 4. Detailed Regression Results
eFigure 2. Co-occurrence of Anxiety and Depression,
eFigure 3. Anxiety and Depression for White Respondents and Individuals From Racially and Ethnically Minoritzed Groups, by Age Group, Annotated With Current Events
eTable 5. Impacts of Pandemic Burden on Anxiety and Depression, Overall and for Ages 18 to 39 Years vs Ages 40 Years and Older
eTable 6. Impacts of Prior COVID-19 Diagnosis and Vaccine Receipt
eFigure 4. Anxiety and Depression by Economic Precarity Score and Age Group
eTable 7. Prevalence of Anxiety and Depression by Economic Precarity
eTable 8. Proportion of Age Disparity Accounted for by Exposure Effect

This supplemental material has been provided by the authors to give readers additional information about their work.

© 2023 Collier Villaume S et al. *JAMA Network Open.*

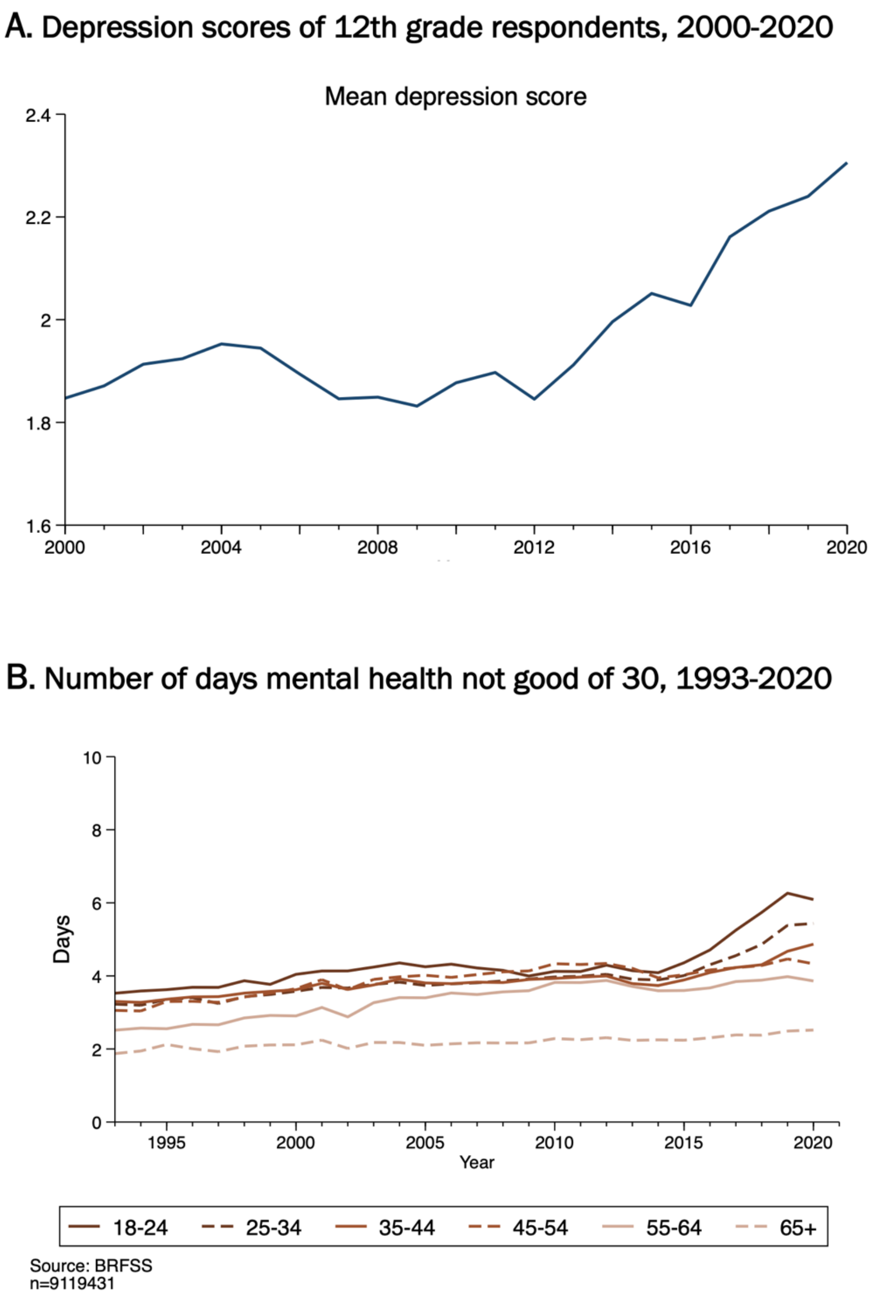

eFigure 1. Pre-pandemic trends in depressive symptoms
Sources: (A) Monitoring the Future, 2000-2020 (authors' calculations); (B) Behavioral Risk Factor Surveillance System, 1993-2020 (authors' calculations).

© 2023 Collier Villaume S et al. *JAMA Network Open.*

eTable 1. Household Pulse Survey administration dates

	HPS Num.	Start date	End date
**Phase 1**	1	April 23, 2020	May 5, 2020
	2	May 7, 2020	May 12, 2020
	3	May 14, 2020	May 19, 2020
	4	May 21, 2020	May 26, 2020
	5	May 28, 2020	June 2, 2020
	6	June 4, 2020	June 9, 2020
	7	June 11, 2020	June 16, 2020
	8	June 18, 2020	June 23, 2020
	9	June 25, 2020	June 30, 2020
	10	July 2, 2020	July 7, 2020
	11	July 9, 2020	July 14, 2020
	12	July 16, 2020	July 21, 2020
**Phase 2**	13	August 19, 2020	August 31, 2020
	14	September 2, 2020	September 14, 2020
	15	September 16, 2020	September 28, 2020
	16	September 30, 2020	October 12, 2020
	17	October 14, 2020	October 26, 2020
**Phase 3**	18	October 28, 2020	November 9, 2020
	19	November 11, 2020	November 23, 2020
	20	November 25, 2020	December 7, 2020
	21	December 9, 2020	December 21, 2020
	22	January 6, 2021	January 18, 2021
	23	January 20, 2021	February 1, 2021
	24	February 3, 2021	February 15, 2021
	25	February 17, 2021	March 1, 2021
	26	March 3, 2021	March 15, 2021
	27	March 17, 2021	March 29, 2021
**Phase 3.1**	28	April 14, 2021	April 26, 2021
	29	April 28, 2021	May 10, 2021
	30	May 12, 2021	May 24, 2021
	31	May 26, 2021	June 7, 2021
	32	June 9, 2021	June 21, 2021
	33	June 23, 2021	July 5, 2021
**Phase 3.2**	34	July 21, 2021	August 2, 2021
	35	August 4, 2021	August 16, 2021
	36	August 18, 2021	August 30, 2021
	37	September 1, 2021	September 13, 2021
	38	September 15, 2021	September 27, 2021

© 2023 Collier Villaume S et al. *JAMA Network Open.*

	39	September 29, 2021	October 11, 2021
**Phase 3.3**	40	December 1, 2021	December 13, 2021
	41	December 29, 2021	January 10, 2022
	42	January 26, 2022	February 7, 2022
**Phase 3.4**	43	March 2, 2022	March 14, 2022
	44	March 30, 2022	April 11, 2022
	45	April 27, 2022	May 9, 2022
**Phase 3.5**	46	June 1, 2022	June 13, 2022
	47	June 29, 2022	July 11, 2022
	48	July 25, 2022	August 8, 2022

Note. Data obtained from Census Bureau
https://www.census.gov/programs-surveys/household-pulse-survey/datasets.html

eTable 2. Descriptive statistics – exposures and covariates

Variable	Percent				OR (95% CI)	
	Overall	18-39	40-59	60+	*18-39 vs. 40-59*	*60+ vs. 40-59*
Female	51.8%	51.2%	51.0%	53.2%	1.01 (.99 to 1.02)	1.09 (1.08 to 1.10)
Age						
18-29	15.9%	-	-	-	-	-
30-39	18.6%	-	-	-	-	-
40-49	17.0%	-	-	-	-	-
50-59	17.3%	-	-	-	-	-
60-69	18.0%	-	-	-	-	-
70 and older	13.0%	-	-	-	-	-
Race/ethnicity						
White (non-Hispanic)	63.6%	57.6%	59.3%	75.0%	.93 (.92 to .95)	2.07 (2.04 to 2.10)
Black (non-Hispanic)	11.0%	10.6%	13.2%	9.1%	.78 (.76 to .80)	.66 (.65 to .67)
Hispanic/Latino (of any race)	16.4%	21.3%	17.7%	9.7%	1.26 (1.23 to 1.28)	.50 (.48 to .51)
Asian or Asian-American	5.2%	6.2%	5.8%	3.4%	1.07 (1.05 to 1.10)	.57 (.56 to .59)
Biracial, multiracial, and all other	3.7%	4.3%	4.0%	2.8%	1.07 (1.04 to 1.10)	.67 (.65 to .70)
BA and above	32.0%	32.8%	33.2%	29.7%	.99 (.97 to 1.00)	.85 (.84 to .86)
Income						
< $25k	15.1%	18.1%	13.1%	14.2%	1.46 (1.43 to 1.49)	1.10 (1.08 to 1.12)
≥ $25 to < 50k	24.1%	25.7%	20.4%	26.7%	1.35 (1.32 to 1.37)	1.42 (1.40 to 1.44)
≥ $50k to < 100k	30.9%	30.2%	29.5%	33.3%	1.03 (1.02 to 1.05)	1.19 (1.18 to 1.21)
≥ $100k	29.8%	26.0%	37.0%	25.8%	**.60 (.59 to .61)**	**.59 (.59 to .60)**
Had COVID	19.7%	23.3%	22.7%	14.2%	1.04 (1.02 to 1.06)	**.57 (.55 to .58)**
Vaccinated (at least one dose)[a]	68.5%	62.7%	69.0%	81.1%	.76 (.74 to .77)	1.93 (1.89 to 1.96)
Live in owned home	69.4%	53.9%	72.0%	83.4%	**0.46 (.45 to .46)**	1.96 (1.93 to 1.99)
Income loss[b]						
since March 2020	47.4%	54.5%	53.2%	32.0%	1.05 (1.03 to 1.07)	.41 (.41 to .42)
in past 4 weeks	15.9%	18.7%	19.1%	9.8%	0.97 (.95 to 1.00)	.46 (.45 to .47)
Worked for pay in last 7 days	57.8%	69.9%	69.6%	31.4%	1.01 (1.00 to 1.03)	.20 (.20 to .20)
Economic adversity score						
0 (most secure)	18.9%	15.1%	20.8%	21.0%	**.68 (.67 to .69)**	1.02 (1.01 to 1.03)
1	33.8%	29.1%	32.8%	40.1%	.84 (.83 to .85)	1.38 (1.36 to 1.39)
2	25.7%	26.2%	25.4%	25.4%	1.04 (1.03 to 1.06)	1.00 (.98 to 1.01)
3	15.4%	19.8%	15.0%	11.1%	1.40 (1.37 to 1.42)	**.71 (.70 to .73)**
4 (most precarious)	6.2%	9.8%	6.0%	2.3%	**1.69 (1.64 to 1.75)**	**.36 (.35 to .38)**

[a] Question added in January 2021(survey 22)
[b] Question wording changed in April 2021 (survey 28)

© 2023 Collier Villaume S et al. *JAMA Network Open.*

eTable 3. Descriptive statistics – outcome variables

Outcome measure	Overall	18-39	40-59	60+
	M(*SD*) or %	*M*(*SD*) or %	*M*(*SD*) or %	*M*(*SD*) or %
Anxiety score (GAD-2)	1.96 (2.04)	2.44 (2.11)	2.00 (2.06)	1.38 (1.79)
Clinically elevated anxiety (score ≥ 3)	30.6%	39.8%	31.2%	19.7%
N	3,022,120	732,665	1,146,794	1,142,661
Depression score (PHQ-2)	1.64 (1.88)	2.05 (2.00)	1.62 (1.87)	1.18 (1.64)
Clinically elevated depression (score ≥ 3)	24.5%	32.6%	24.0%	16.1%
N	3,019,886	732,105	1,145,993	1,141,788

Note. Mean and standard deviation are presented for anxiety and depression scores. Percentages reflect the proportion of participants with scores that exceed thresholds for clinical significance. *N* corresponds to the number of participants with complete data for each outcome. Population weights are applied.

© 2023 Collier Villaume S et al. *JAMA Network Open.*

eTable 4. Detailed regression results

	Full dataset		Respondents with income data	
	Anxiety	Depression	Anxiety	Depression
	(1)	(2)	(3)	(4)
Female	8.43 (8.19 to 8.67)	3.85 (3.62 to 4.08)	7.09 (6.84 to 7.34)	2.40 (2.16 to 2.64)
Race/ethnicity				
Black (non-Hispanic)	-1.07 (-1.51 to -0.63)	1.40 (0.98 to 1.83)	-4.23 (-4.70 to -3.76)	-2.00 (-2.45 to -1.55)
Hispanic/Latino (of any race)	-0.90 (-1.34 to -0.46)	0.05 (-0.37 to 0.48)	-3.60 (-4.06 to -3.14)	-2.94 (-3.38 to -2.49)
Asian or Asian-American	-8.63 (-9.15 to -8.1)	-3.58 (-4.08 to -3.08)	-9.31 (-9.84 to -8.77)	-4.21 (-4.74 to -3.69)
Biracial, multiracial, and all other	5.70 (5.06 to 6.34)	6.51 (5.87 to 7.16)	4.12 (3.46 to 4.78)	4.71 (4.04 to 5.37)
BA and above	-6.32 (-6.53 to -6.11)	-9.61 (-9.81 to -9.42)	-1.86 (-2.09 to -1.64)	-4.82 (-5.03 to -4.6)
Age				
30-39	-4.71 (-5.21 to -4.22)	-7.61 (-8.10 to -7.12)	-3.72 (-4.24 to -3.19)	-6.40 (-6.92 to -5.89)
40-49	-8.79 (-9.28 to -8.3)	-11.47 (-11.95 to -10.99)	-6.83 (-7.35 to -6.3)	-9.28 (-9.79 to -8.77)
50-59	-13.09 (-13.57 to -12.6)	-13.82 (-14.29 to -13.34)	-11.29 (-11.81 to -10.78)	-11.71 (-12.22 to -11.21)
60-69	-20.23 (-20.70 to -19.76)	-18.94 (-19.40 to -18.48)	-19.50 (-20.00 to -18.99)	-17.92 (-18.41 to -17.43)
70 and older	-26.67 (-27.17 to -26.17)	-23.33 (-23.82 to -22.84)	-26.99 (-27.52 to -26.46)	-23.44 (-23.96 to -22.92)
Income				
≥ $25 to < 50k			-7.13 (-7.63 to -6.63)	-7.83 (-8.32 to -7.34)
≥ $50k to < 100k			-13.05 (-13.52 to -12.58)	-14.56 (-15.02 to -14.1)
≥ $100k			-19.62 (-20.10 to -19.15)	-21.04 (-21.50 to -20.58)
Intercept	40.40 (39.51 to 41.29)	36.28 (35.43 to 37.13)	50.98 (50.00 to 51.96)	47.7 (46.76 to 48.65)
State fixed effects	X	X	X	X
Survey week fixed effects	X	X	X	X
Controls for income			X	X
N	3,022,120	3,019,886	2,770,119	2,769,229

Note. Each model presents results from a separate multiple regression model that predicts anxiety or depression from a set of covariates and fixed effects. Cells contain regression coefficients, with 95% confidence intervals in parenthesis. The coefficients reflect estimates of population prevalence (such that “40.0” corresponds to 40% of the population).

© 2023 Collier Villaume S et al. *JAMA Network Open.*

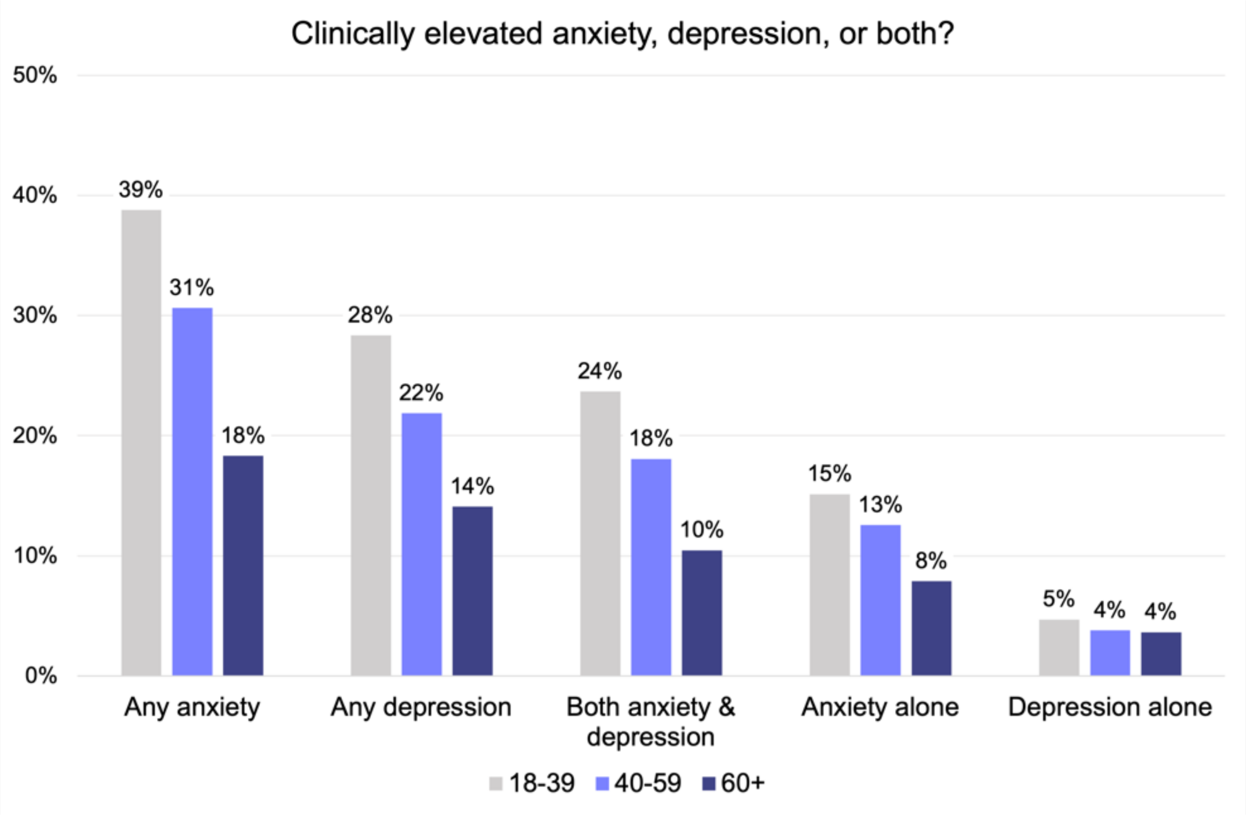

eFigure 2. Co-occurrence of anxiety and depression
Source: Household Pulse Surveys, April 2020 – August 2022 (authors' calculations)

© 2023 Collier Villaume S et al. *JAMA Network Open.*

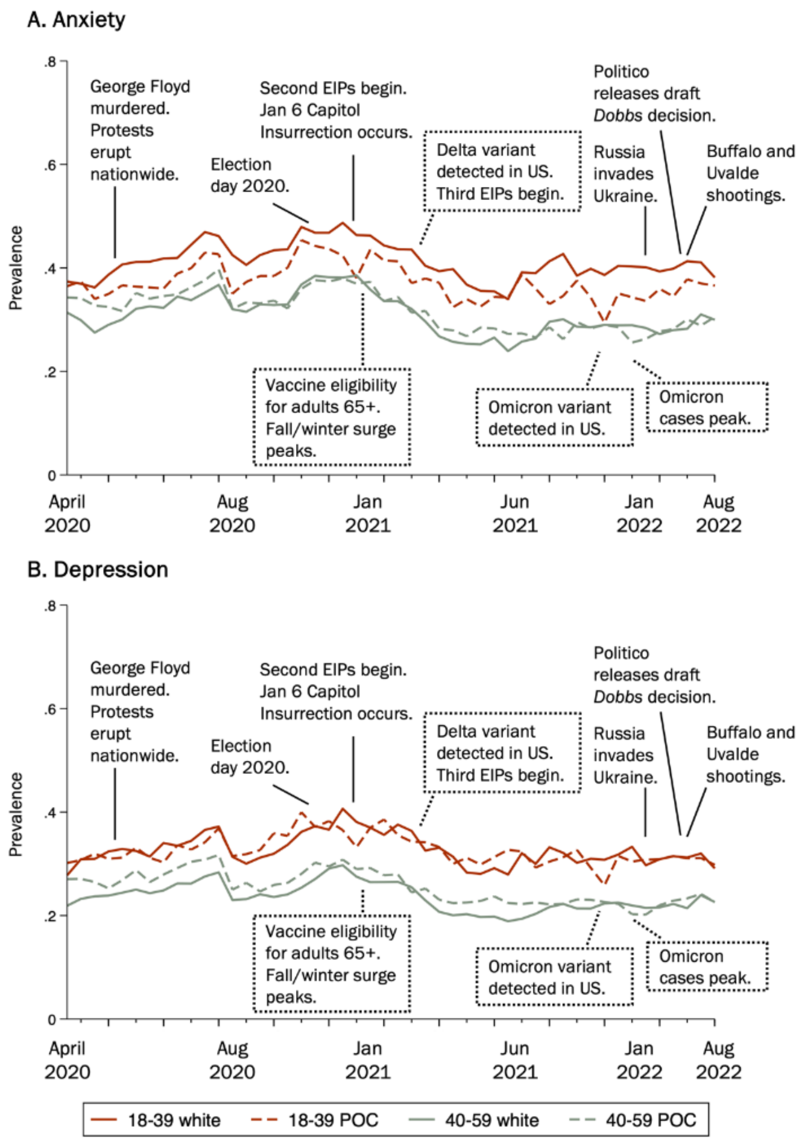

eFigure 3. Anxiety and Depression for White Respondents and Individuals From Racially and Ethnically Minoritzed Groups, by Age Group, Annotated With Current Events

Source: Household Pulse Surveys, April 2020-August 2022 (authors' calculations)

Note. This figure shows that white respondents exhibit a larger spread in anxiety and depression than people of color (POC), with the highest levels of anxiety and depression among white respondents 18-39 and the lowest levels among white respondents 40-59 (whereas prevalence for POC tends to fall between these extremes for both age groups). Annotations provide information about contemporaneous events and should not be interpreted as suggesting causal associations. Plots reflect population-weighted descriptive statistics (not regression-adjusted values). Dashed boxes signify current events related to the coronavirus pandemic.
EIP = "Economic Impact Payment."

© 2023 Collier Villaume S et al. *JAMA Network Open.*

eTable 5. Impacts of pandemic burden on anxiety and depression, overall and for 18-39 vs 40+

	Full survey period		*April-December 2020*	*January 2021 - August 2022*
	(1)	(2)	(3)	(4)
Anxiety				
Cases	**4.03 (1.93 to 6.13)*****	**3.05 (0.76 to 5.34)***	7.27 (-0.14 to 14.67) †	**4.46 (1.67 to 7.25)****
Cases^2	**-1.31 (-2.42 to -0.20)***	-1.01 (-2.24 to .21)	-4.67 (-13.77 to 4.43)	**-1.82 (-3.18 to -0.46)****
Cases*under40		1.98 (-0.60 to 4.56)	**9.08 (0.45 to 17.71)***	-2.55 (-5.74 to 0.65)
Cases^2*under40		-0.63 (-2.29 to 1.03)	-8.28 (-20.62 to 4.07)	1.48 (-0.42 to 3.38)
Deaths	**20.63 (8.22 to 33.04)****	**28.37 (14.44 to 42.30)*****	**34.96 (5.72 to 64.20)***	**21.74 (3.66 to 39.83)***
Deaths^2	-40.8 (-90.20 to 8.61)	-15.51 (-33.41 to 2.40) †	-80.58 (-252.45 to 91.30)	-62.71 (-127.12 to 1.70) †
Deaths*under40		**-74.91 (-132.05 to -17.77)***	-4.64 (-41.84 to 32.56)	**-22.15 (-43.74 to -0.56)***
Deaths^2*under40		68.76 (-16.25 to 153.77)	7.3 (-223.26 to 237.85)	81.04 (-10.89 to 172.97) †
Depression				
Cases	**3.79 (1.76 to 5.82)*****	**2.78 (0.59 to 4.96)***	6.4 (-0.72 to 13.51) †	**3.00 (0.35 to 5.65)***
Cases^2	**-1.57 (-2.62 to -0.51)****	**-1.43 (-2.57 to -0.28)***	-0.22 (-8.89 to 8.45)	**-1.78 (-3.06 to -0.50)****
Cases*under40		2.07 (-0.43 to 4.57)	**12.30 (3.95 to 20.66)****	-1.81 (-4.90 to 1.28)
Cases^2*under40		-0.32 (-1.92 to 1.27)	**-13.75 (-25.76 to -1.73)***	1.53 (-0.29 to 3.36)
Deaths	**23.58 (11.47 to 35.70)*****	**23.86 (10.46 to 37.26)*****	**61.33 (33.31 to 89.35)*****	12.59 (-4.81 to 30.00)
Deaths^2	-43.71 (-92.68 to 5.25) †	-51.46 (-107.02 to 4.10) †	**-234.1 (-393.41 to -74.78)****	-19.76 (-82.28 to 42.77)
Deaths*under40		-0.57 (-18.10 to 16.97)	-7.24 (-43.24 to 28.76)	-2.32 (-23.43 to 18.79)
Deaths^2*under40		15.80 (-68.95 to 100.55)	53.82 (-163.79 to 271.42)	13.96 (-77.57 to 105.49)

Note. † $p < .10$, * $p < .05$, ** $p < .01$, *** $p < .001$. Cells contain regression coefficients, with 95% confidence intervals in parenthesis. Coronavirus cases (per 100 state residents) and deaths (per 1,000 state residents) are each modeled in separate regressions predicting anxiety or depression. Covariates include sex, ethnoracial group, education level, 10-year age category, and state and survey-week fixed effects. Sample is restricted to adults ages 18-59. Columns 1 and 2 contain results for all available survey weeks (surveys 1-48). The survey period is then split into two timeframes, with results from surveys fielded in 2020 (surveys 1-21; corresponds to the period that preceded coronavirus vaccines) in column 3 and those in 2021 and 2022 (surveys 22-48) in column 4.

© 2023 Collier Villaume S et al. *JAMA Network Open.*

eTable 6. Impacts of prior COVID-19 diagnosis and vaccine receipt

	Overall		With under40 interaction	
	Coef (95% CI)	*p*-value	Coef (95% CI)	*p*-value
Anxiety				
Had COVID	0.47 (-0.04 to 0.99)	0.07	0.89 (0.28 to 1.51)	0.004
Had COVID x under40			-0.86 (-1.85 to 0.12)	0.09
Vaccinated	0.17 (-0.38 to 0.73)	0.54	-2.05 (-2.68 to -1.42)	< .001
Vaccinated x under40			4.29 (3.41 to 5.17)	< .001
Depression				
Had COVID	-0.34 (-0.84 to 0.16)	0.18	0.32 (-0.26 to 0.90)	0.29
Had COVID x under40			-1.34 (-2.29 to -0.39)	0.006
Vaccinated	-0.82 (-1.36 to -0.28)	0.003	-2.17 (-2.77 to -1.57)	< .001
Vaccinated x under40			2.60 (1.75 to 3.45)	< .001

Note. Prior infection ("Had COVID") and vaccination are each modeled in separate regressions predicting anxiety and depression. Covariates include sex, ethnoracial group, education level, 10-year age category, and state and survey-week fixed effects. Sample is restricted to adults ages 18-59. Both survey items were added to the HPS in January 2021.

© 2023 Collier Villaume S et al. *JAMA Network Open.*

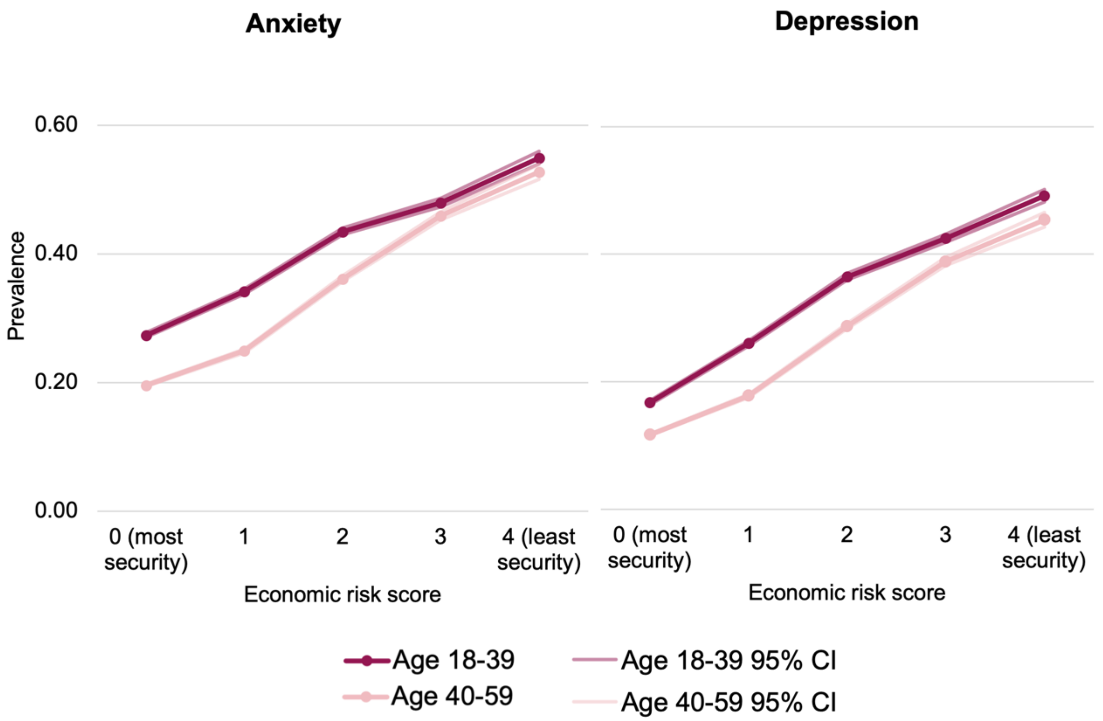

eFigure 4. Anxiety and depression by economic precarity score and age group
Source: Household Pulse Surveys, April 2020 – August 2022 (authors' calculations).

© 2023 Collier Villaume S et al. *JAMA Network Open.*

eTable 7. Prevalence of anxiety and depression by economic precarity

	Overall		With under40 interaction	
	Coef (95% CI)	*p*-value	Coef (95% CI)	*p*-value
Anxiety				
Home owned	-5.28 (-5.67 to -4.88)	< .001	-7.05 (-7.57 to -6.54)	< .001
Home owned x under40			3.18 (2.47 to 3.89)	< .001
Employed	-8.6 (-9 to -8.2)	< .001	-11.83 (-12.31 to -11.34)	< .001
Employed x under40			6.54 (5.78 to 7.3)	< .001
Income loss due to pandemic	14.25 (-13.83 to -14.66)	< .001	15.23 (14.72 to 15.74)	< .001
Income loss (past month)	18.80 (-18.07 to -19.53)	< .001	19.41 (18.53 to 20.30)	< .001
Income loss due to pandemic x under40			-1.95 (-2.67 to -1.23)	< .001
Income loss (past month) x under40			-1.30 (-2.74 to 0.13)	0.075
Risk score 1	9.97 (9.6 to 10.33)	< .001	9.6 (9.18 to 10.02)	< .001
Risk score 2	21.12 (20.58 to 21.67)	< .001	21.39 (20.76 to 22.02)	< .001
Risk score 3	28.15 (27.37 to 28.93)	< .001	31 (30.1 to 31.91)	< .001
Risk score 4	35.27 (34.2 to 36.34)	< .001	38.14 (36.76 to 39.52)	< .001
Risk score 1 x under40			0.42 (-0.24 to 1.08)	0.22
Risk score 2 x under40			-1.05 (-1.86 to -0.24)	< .001
Risk score 3 x under40			-5.72 (-6.75 to -4.7)	< .001
Risk score 4 x under40			-5.41 (-6.97 to -3.86)	< .001
Depression				
Home owned	-4.6 (-4.98 to -4.22)	< .001	-6.03 (-6.53 to -5.54)	< .001
Home owned x under40			2.58 (1.89 to 3.26)	< .001
Employed	-9.29 (-9.68 to -8.9)	< .001	-11.71 (-12.18 to -11.24)	< .001
Employed x under40			4.9 (4.16 to 5.64)	< .001
Income loss due to pandemic	11.84 (11.43 to 12.24)	< .001	11.83 (11.35 to 12.32)	< .001
Income loss (past month)	16.00 (15.28 to 16.72)	< .001	15.71 (14.84 to 16.57)	< .001
Income loss (past month) x under40			0.01 (-0.70 to 0.71)	0.99
Income loss due to pandemic x under40			0.63 (-0.79 to 2.04)	0.39
Risk score 1	7.49 (7.16 to 7.82)	< .001	6.43 (6.05 to 6.81)	< .001
Risk score 2	17.14 (16.63 to 17.66)	< .001	16.26 (20.58 to 21.67)	< .001
Risk score 3	23.62 (22.87 to 24.37)	< .001	24.82 (15.67 to 16.85)	< .001
Risk score 4	29.75 (28.7 to 30.8)	< .001	30.93 (29.58 to 32.28)	< .001
Risk score 1 x under40			2.11 (1.51 to 2.71)	< .001
Risk score 2 x under40			1.57 (0.81 to 2.32)	< .001
Risk score 3 x under40			-2.27 (-3.25 to -1.29)	< .001
Risk score 4 x under40			-2.01 (-3.55 to -0.48)	0.01

Note. Each intendent variable (home ownership, income loss, etc.) is modeled here in a separate regression predicting anxiety or depression. Shading separates one set of models from the next. Covariates include sex, ethnoracial group, education level, 10-year age category, income and state and survey-week fixed effects. Sample is restricted to adults ages 18-59.

© 2023 Collier Villaume S et al. *JAMA Network Open.*

eTable 8. Proportion of age disparity accounted for by exposure effect

	A. Base models		B. Including measures of economic precarity			
	(1)	(2)	(3)	(4)	(5)	(6)
Anxiety	0.222	0.047	0.314	0.217	0.198	0.365
% chg vs Model 1		*-78.85%*	*41.13%*	*-2.35%*	*-10.77%*	*64.22%*
Depression	0.221	0.033	0.307	0.215	0.204	0.354
% chg vs Model 1		*-84.90%*	*38.77%*	*-2.63%*	*-7.82%*	*60.20%*
Demographic variables[1]	X	X	X	X	X	X
Income	X		X	X	X	X
Home owned			X			
Recent employment				X		
Income loss					X	
Risk score						X
COVID Cases						
Had COVID						
Vaccinated						
Timeframe						
Weeks 1-48	X	X	X	X	X	X
Weeks 22-48						

	C. Including pandemic-related exposures					
	(7)	(8)	(9)	(10)	(11)	(12)
Anxiety	0.222	0.231	0.226	0.209	0.183	0.185
% chg vs Model 8			*-1.90%*	*-9.27%*	*-20.79%*	*-20.06%*
Depression	0.221	0.232	0.227	0.208	0.189	0.189
% chg vs Model 8			*-2.17%*	*-10.61%*	*-18.81%*	*-18.78%*
Demographic variables[1]	X	X	X	X	X	X
Income	X	X	X	X	X	X
Home owned						
Recent employment						
Income loss						
Risk score						
COVID Cases	X		X	X	X	X
Had COVID				X		X
Vaccinated					X	X
Timeframe						
Weeks 1-48	X					
Weeks 22-48		X	X	X	X	X

Note. Each column contains a numeric result for anxiety (top row) and depression. Numeric results reflect the *share* of the difference between the two groups (18-39 and 40+) that is attributed to the endowment effect. Below the horizontal line, Xs indicate whether a given covariate is included and the timeframe that each model covers.

© 2023 Collier Villaume S et al. *JAMA Network Open.*
