# Supplementary material for: Age Disparities in Prevalence of Anxiety and Depression Among US Adults During the COVID-19 Pandemic
Source: JAMA Netw Open. 2023 Nov 30;6(11):e2345073. doi: 10.1001/jamanetworkopen.2023.45073 (PMC10690464; doi:10.1001/jamanetworkopen.2023.45073)
Supplement: Supplement 2. — Data Sharing Statement [file jamanetwopen-e2345073-s002.pdf]

Data Sharing Statement

Data
Data available: Yes
Data types: Deidentified participant data
How to access data: https://github.com/colliesa/there-it-is-again
When available: With publication

Supporting Documents
Document types: Statistical/analytic code
How to access documents: https://github.com/colliesa/there-it-is-again
When available: With publication

Additional Information
Who can access the data: Github repository will be made publicly available.
Types of analyses: For any purpose
Mechanisms of data availability: Github repository will be made publicly available.
